# A Comprehensive Analysis of Shared Loci between Systemic Lupus Erythematosus (SLE) and Sixteen Autoimmune Diseases Reveals Limited Genetic Overlap

**DOI:** 10.1371/journal.pgen.1002406

**Published:** 2011-12-08

**Authors:** Paula S. Ramos, Lindsey A. Criswell, Kathy L. Moser, Mary E. Comeau, Adrienne H. Williams, Nicholas M. Pajewski, Sharon A. Chung, Robert R. Graham, Raphael Zidovetzki, Jennifer A. Kelly, Kenneth M. Kaufman, Chaim O. Jacob, Timothy J. Vyse, Betty P. Tsao, Robert P. Kimberly, Patrick M. Gaffney, Marta E. Alarcón-Riquelme, John B. Harley, Carl D. Langefeld, for The International Consortium on the Genetics of Systemic Erythematosus (SLEGEN)

**Affiliations:** 1 Department of Medicine, Medical University of South Carolina, Charleston, South Carolina, United States of America; 2 Rosalind Russell Medical Research Center for Arthritis, University of California San Francisco, San Francisco, California, United States of America; 3 Arthritis and Immunology Research Program, Oklahoma Medical Research Foundation, Oklahoma City, Oklahoma, United States of America; 4 Department of Biostatistical Sciences, Wake Forest University Health Sciences, Winston-Salem, North Carolina, United States of America; 5 Immunology Biomarkers Group, Genentech, South San Francisco, California, United States of America; 6 Department of Cell Biology and Neuroscience, University of California Riverside, Riverside, California, United States of America; 7 Division of Rheumatology, Cincinnati Children's Hospital Medical Center, Cincinnati, Ohio, United States of America; 8 Department of Medicine, Keck School of Medicine, University of Southern California, Los Angeles, California, United States of America; 9 Division of Genetics and Molecular Medicine, King's College London, London, United Kingdom; 10 David Geffen School of Medicine, University of California Los Angeles, Los Angeles, California, United States of America; 11 Department of Medicine, University of Alabama at Birmingham, Birmingham, Alabama, United States of America; 12 Centro de Genómica y Investigaciones Oncológicas, Pfizer-Universidad de Granada-Junta de Andalucía, Granada, Spain; University of Geneva Medical School, Switzerland

## Abstract

In spite of the well-known clustering of multiple autoimmune disorders in families, analyses of specific shared genes and polymorphisms between systemic lupus erythematosus (SLE) and other autoimmune diseases (ADs) have been limited. Therefore, we comprehensively tested autoimmune variants for association with SLE, aiming to identify pleiotropic genetic associations between these diseases. We compiled a list of 446 non–Major Histocompatibility Complex (MHC) variants identified in genome-wide association studies (GWAS) of populations of European ancestry across 17 ADs. We then tested these variants in our combined Caucasian SLE cohorts of 1,500 cases and 5,706 controls. We tested a subset of these polymorphisms in an independent Caucasian replication cohort of 2,085 SLE cases and 2,854 controls, allowing the computation of a meta-analysis between all cohorts. We have uncovered novel shared SLE loci that passed multiple comparisons adjustment, including the *VTCN1* (rs12046117, P = 2.02×10^−06^) region. We observed that the loci shared among the most ADs include *IL23R*, *OLIG3/TNFAIP3*, and *IL2RA*. Given the lack of a universal autoimmune risk locus outside of the MHC and variable specificities for different diseases, our data suggests partial pleiotropy among ADs. Hierarchical clustering of ADs suggested that the most genetically related ADs appear to be type 1 diabetes with rheumatoid arthritis and Crohn's disease with ulcerative colitis. These findings support a relatively distinct genetic susceptibility for SLE. For many of the shared GWAS autoimmune loci, we found no evidence for association with SLE, including *IL23R*. Also, several established SLE loci are apparently not associated with other ADs, including the *ITGAM-ITGAX* and *TNFSF4* regions. This study represents the most comprehensive evaluation of shared autoimmune loci to date, supports a relatively distinct non–MHC genetic susceptibility for SLE, provides further evidence for previously and newly identified shared genes in SLE, and highlights the value of studies of potentially pleiotropic genes in autoimmune diseases.

## Author Summary

It is well known that multiple autoimmune disorders cluster in families. However, all of the genetic variants that explain this clustering have not been discovered, and the specific genetic variants shared between systemic lupus erythematosus (SLE) and other autoimmune diseases (ADs) are not known. In order to better understand the genetic factors that explain this predisposition to autoimmunity, we performed a comprehensive evaluation of shared autoimmune genetic variants. First we considered results from 17 ADs and compiled a list with 446 significant genetic variants from these studies. We identified some genetic variants extensively shared between ADs, as well as the ADs that share the most variants. The genetic overlap between SLE and other ADs was modest. Next we tested how important all the 446 genetic variants were in our collection with a minimum of 1,500 SLE patients. Among the most significant variants in SLE, the majority had already been identified in previous studies, but we also discovered variants in two important immune genes. In summary, our data identified diseases with common genetic risk factors and novel SLE effects, and this supports a relatively distinct genetic susceptibility for SLE. This study helps delineate the genetic architecture of ADs.

## Introduction

Systemic lupus erythematosus (SLE [MIM 152700]) is a chronic and severe systemic autoimmune disease characterized by the production of high titers of autoantibodies directed against native DNA and other cellular constituents. It is a prototypic autoimmune disease with heterogeneous clinical manifestations that may involve many different organs and tissues, including skin, kidney, lungs, heart, and brain. The prevalence of SLE in the U.S. is estimated to be between 0.05% and 0.1% of the population, disproportionately affecting women and African Americans (0.009% of white men, 0.066% of white women, 0.038% of African-American men, and 0.282% of African-American women) [1]. A genetic etiology for SLE is unequivocal, as recent genome-wide association studies (GWAS) have identified nearly 40 validated susceptibility loci and implicated a broad array of biological pathways [2]. Nevertheless, recent estimates suggest that these risk loci collectively explain between 8%–15% of the genetic risk for SLE [3], [4], highlighting the fact that much of the heritable basis for SLE remains to be identified.

The clustering of multiple autoimmune diseases (ADs) within families, including families with SLE [5], [6], suggests some degree of common genetic susceptibility [7]–[9]. This genetic overlap is exemplified by the well-known associations of certain Human Leukocyte Antigen (HLA) loci with multiple human ADs, as well as non-HLA risk loci in diverse pathways such as *IL2RA*, *STAT4*, *PTPN22* and *IFIH1* [10]. This phenomenon where a single mutation or gene can affect multiple traits is known as pleiotropy. Murine studies have similarly identified many susceptibility loci that are shared across different autoimmune mouse models [11]. However, evidence for specific shared risk variants is modest, and consequently the genetic mechanisms that may explain the patterns of disease aggregation remain unclear.

To date, there is no large-scale, comprehensive assessment of the genetic overlap between SLE and other ADs. Multiple genes have been reported to be associated with both SLE and other ADs, but analyses of such shared autoimmune loci have been limited to specific loci and few diseases (reviewed in [12]). Criswell *et al.* [13] analyzed a collection of 265 multiplex families with at least two ADs. Based on findings concerning *PTPN22*, they suggest that multiple sclerosis (MS) may have a pathogenesis that is distinct from SLE, rheumatoid arthritis (RA) and type 1 diabetes (T1D).

Several genome-wide association studies (GWAS) have been conducted in multiple ADs, providing an opportunity to assess genetic similarity at the genome-wide scale. These include studies of SLE, RA, T1D, MS, ankylosing spondylitis (AS), inflammatory bowel disease (IBD), Crohn's disease (CD), ulcerative colitis (UC), celiac disease (CelD), psoriasis (PS), psoriatic arthritis (PsA), juvenile idiopathic arthritis (JIA), Kawasaki disease (KA), systemic sclerosis (SScl), sarcoidosis (SA), vitiligo (VI), alopecia areata (AA) and Behçet's disease (BeD). Several studies have evaluated pleiotropic effects between two or three diseases, but have been limited to a few dozen variants in a few loci [14]–[18]. Exceptions include that of Sirota *et al.* [19], which used over 500 SNPs to analyze allele-specific similarities and differences across six ADs, and Thompson *et al.* [20], which evaluated the association of over 500 reported autoimmune loci with JIA. Wang *et al.* [21] similarly performed a genome-wide comparative analysis of CD, UC and T1D, and Festen *et al.* [22] of CD and CelD. Only Cotsapas et al. [23] have recently analyzed shared variation of 107 immune SNPs between seven Ads including SLE.

In order to assess the genetic overlap between SLE and other ADs, potentially unveiling novel contributors to SLE pathogenesis, we comprehensively tested all non-HLA variants implicated in other ADs through large GWA approaches with P<1.0×10^−5^, in a large SLE cohort consisting of 1500 cases and 5706 controls [24]–[27]. The primary advantages of this approach include the opportunity to identify consistent or contrasting allelic risk, the potential to more clearly identify common pathways, and a more focused, narrow hypothesis space that generates more statistical power due to fewer statistical tests. We did not include MHC variants because they were not reported in all the GWAS. The comparison and contrast of shared and distinct AD risk loci provide the potential to improve diagnosis and prognosis and help identify plausible pharmacological targets. Our data suggests that, compared to other ADs, SLE exhibits modest overlap of associated loci with other ADs. We have also uncovered novel shared SLE loci. This study helps better understand the non-MHC genetic architecture of ADs. Identification of novel SLE genes and shared genetic pathways can contribute to a better understanding of common genetic mechanisms, and eventually the development of improved diagnosis, prognosis and targeted therapies.

## Results

We compiled a list with 446 non-Major Histocompatibility Complex (MHC) variants identified as significant in 74 Caucasian GWAS of 17 autoimmune diseases (ADs) (Table 1) using a publically available database [28]. Please see Materials and Methods for further details regarding the selection of the AD variants. Note that for the results herein discussed, we have excluded loci reported from joint analyses of particular ADs, such as the combined phenotype of inflammatory bowel disease (see Materials and Methods). While this may exclude legitimate shared risk loci, it was done so as not to structurally impose a greater degree of genetic overlap amongst the handful of ADs that have been directly analyzed together. Based on a block partitioning approach using LD estimated from the CEU sample of the International HapMap Project, we then mapped these SNPs to 337 genomic regions.

**Table 1:** Data compiled from published GWA studies of autoimmune diseases (ADs).

Disease	Number of		
	Associated loci	Associated SNPs	GWAS
Alopecia areata (AA)	7	7	1
Ankylosing spondylitis(AS)	8	8	1
Juvenile idiopathic arthritis (JIA)	1	1	1
Behcet's disease (BeD)	2	2	1
Celiac disease (CelD)	42 (53)	42 (55)	3 (5)
Crohn's disease (CD)	79 (84)	103 (111)	9 (14)
Kawasaki disease (KA)	2	2	1
Multiple sclerosis(MS)	37	41	9
Psoriasis (PS)	27	30	6
Psoriatic arthritis (PsA)	2	2	1
Rheumatoid arthritis (RA)	39 (48)	42 (55)	7 (8)
Sarcoidosis (SA)	1	1	1
Systemic lupus erythematosus (SLE)	31	36	5
Systemic sclerosis (SScl)	3	3	1
Type 1 diabetes (T1D)	56	64	8
Ulcerative colitis (UC)	59 (64)	77 (86)	6 (9)
Vitiligo (VI)	12	12	2

Counts in parentheses include GWAS of combined ADs including inflammatory bowel disease (CD+UC), CelD+RA, CD+CelD, and CD+SA (see Materials and Methods).

In order to identify novel SLE loci and assess the extent of pleiotropy between SLE and other ADs, we tested the 446 aforementioned non-MHC variants for association with SLE in a large cohort of 1500 SLE cases and 5706 controls. This cohort consists of the joint-analysis of previously described cohorts [24]–[27], which haven't previously been analyzed together. Of the 424 available SNPs based on direct genotyping or imputation, 237 (55.9%) met all quality control criteria. We employed a simple strategy to address the multiple testing issue: we computed a False Discovery Rate (FDR)-adjusted P-value [28] in joint analysis results (P_FDR_), and discarded any variants that did not meet a FDR-adjusted P-value (that is, P_FDR_≥0.05) as likely false positives. Therefore, we herein report the unadjusted *P-values* in the joint-analysis and only report variants that survived a FDR [29] correction for the number of comparisons in the joint-analysis (P_FDR_). We have also elected to report the genomic control (GC)-adjusted P-value. Of the 237 SNPs that met quality control (QC) criteria, 39 survived a FDR adjustment with P<0.05. If a variant failed quality control criteria in the joint-analysis, but met them in the Lupus Large Association Study (LLAS) replication cohort, we report the LLAS cohort results, as indicated in the tables. Finally, if a SNP met quality control criteria in all cohorts, we additionally report the meta-analysis results.

### Novel SLE loci

Our first goal was the identification of novel pleiotropic regions associated with SLE. An intronic variant in the *V-set domain containing T cell activation inhibitor 1* (*VTCN1*) showed the smallest P-value in the joint-analysis (rs12046117, P = 2.02×10^−06^, P_FDR_ = 5.33×10^−05^) (Table 2). This variant was one of the most significant reported in a GWAS of JIA [28]. Unfortunately, the risk allele was not reported.

**Table 2:** Association results in novel pleiotropic regions associated with SLE.

					Joint-Analysis					Meta-Analysis			
SNP^1^	Ch	Pos (Mb)	MAF case	MAF control	MA	P-value^2^	GC P-value	FDR P-value	OR [95%CI]	P-value^2^	OR [95%CI]	Region	Diseases associated with region in GWAS^3^
rs12046117^SMU^	1	117.463	0.07	0.01	T	2.02E-06^d^	2.15E-06	5.33E-05	1.65[1.34–2.03]	NA	NA	VTCN1	JIA
rs6738825^SMU^	2	198.722	0.45	0.48	G	3.12E-03^d^	3.32E-03	2.12E-02	0.82[0.72–0.94]	NA	NA	PLCL1	CD
rs17810546^M^	3	161.148	0.10	0.11	G	2.47E-03	2.63E-03	1.89E-02	0.82[0.72–0.93]	9.39E-03	0.89[0.81–0.97]	IL12A	MS, CelD
rs7672826	4	182.775	0.31	0.33	G	1.90E-03^d^	2.02E-03	1.56E-02	0.83[0.74–0.93]	NA	NA	RPL19P8	MS
rs881375^SMU^	9	120.732	0.36	0.33	T	3.89E-03	4.14E-03	2.49E-02	1.14[1.04–1.24]	NA	NA	VEGFA	UC, CD
rs1953126^SMU^	9	120.720	0.36	0.33	T	2.93E-03^d^	3.12E-03	2.17E-02	1.20[1.06–1.35]	NA	NA	TRAF1	RA, CelD
rs7221109^SU^	17	36.024	0.41	0.38	T	2.21E-03^d^	2.35E-03	1.74E-02	1.21[1.07–1.36]	NA	NA	CCR7	T1D
rs6074022^SM^	20	44.174	0.28	0.26	C	1.41E-03^r^	1.50E-03	1.24E-02	1.45[1.15–1.82]	NA	NA	CD40	RA, MS
rs2297441^SU^	20	61.798	0.20	0.23	G	7.63E-04	8.11E-04	8.22E-03	0.84[0.76–0.93]	NA	NA	ZGPAT	UC, CD

Only the most significant variant in each region is presented. All variants met a FDR-adjusted threshold of significance in the joint-analysis (FDR P<0.05), as described in the Materials and Methods. The genomic control-adjusted (GC) P-value is also shown. When both joint and LLAS1 results available, a meta-analysis between all cohorts is presented. The smallest *P-value* is presented and, unless noted otherwise, it is under the additive model. OR and CI calculated under the model presented.

Ch – chromosome; Mb – Megabases; MA – minor allele; MAF – Minor allele frequency; OR – odds ratio; CI – confidence interval; NA – not available.

1 The initials after the marker denote that this marker was imputed in a cohort: ^S^SLEGEN, ^M^MN, ^U^UCSF.

2 The superscript after the P-value denotes its genetic model, when other than the additive: ^d^dominant, ^r^recessive.

3 Diseases that reported any associated SNP with P-value<1.0×10^−5^ in the regions indicated (not necessarily the same SNP reported in this table) through the GWA approach [28].

Disease abbreviations are the same as for Table 1.

We have also observed association with the *zinc finger, CCCH-type with G patch domain* (*ZGPAT*) region, identified in GWAS of CD and UC [28]. The most significant SNP (rs2297441, P = 7.63×10^−04^, P_FDR_ = 8.22×10^−03^) (Table 2) is located in both the 5′ UTR of the *tumor necrosis factor receptor superfamily member 6B* (*TNFRSF6B*) and the 3′ UTR of the *regulator of telomere elongation helicase 1* (*RTEL1*). The risk allele is the same as the one reported in UC.

The *CD40* gene, which was reported as significant in GWAS of MS and RA [28], showed evidence for association at several variants in linkage disequilibrium (LD) (r^2^>0.70), the most significant being rs6074022 (P = 1.41×10^−03^, P_FDR_ = 1.24×10^−02^) (Table 2), upstream of *CD40*. The risk allele for this variant is the same in SLE as the one reported in MS. All the variants lie within a known CNV region.

The *IL12A* region, identified in GWAS of MS and CelD [28], showed association with SLE at two variants not in LD with each other (r^2^ = 0.04). The most significant association was with rs17810546 (P_meta_ = 9.39×10^−03^) (Table 2), upstream of *IL12A*, but the risk allele is different from that reported in CeID.

### Shared SLE loci

One of our goals was to specifically evaluate pleiotropy between SLE and other ADs. In Table 3 we report our most significant findings in regions previously reported to be associated with SLE. The ADs that reported a GWA P<1.0×10^−5^ at any variant in these regions are shown.

**Table 3:** Shared loci: Association results for SLE susceptibility loci associated with another autoimmune disease.

					Joint-Analysis*					Meta-Analysis			
SNP^1^	Ch	Pos (Mb)	MAF case	MAF control	MA	P-value^2^	GC P-value	FDR P-value	OR[95%CI]	P-value^2^	OR[95%CI]	Region	Diseases associated with region in GWAS^3^
rs10800309*	1	159.739	0.30	0.33	A	5.03E-05	5.35E-05	NA	0.85[0.78–0.92]	NA	NA	FCGR2A	UC
rs3024493^SMU^	1	205.011	0.18	0.15	C	4.38E-05	4.66E-05	6.48E-04	1.26[1.13–1.41]	NA	NA	IL10	T1D, CD, UC, BeD
rs6445975^M^	3	58.345	0.32	0.29	T	4.51E-04	4.79E-04	5.09E-03	1.18[1.07–1.29]	5.27E-09	1.20[1.13–1.27]	PXK	SLE
rs13119723^MU^	4	123.438	0.11	0.13	G	5.56E-03^d^	5.91E-03	3.47E-02	0.82[0.71–0.94]	4.97E-05^d^	0.83[0.76–0.91]	KIAA1109	RA, T1D, CelD
rs11747270^SMU^	5	150.239	0.05	0.02	G	1.12E-03^d^	1.19E-03	1.15E-02	1.39[1.14–1.70]	NA	NA	IRGM	CD
rs5029939^S^	6	138.237	0.06	0.03	G	1.51E-14^d^	1.61E-14	8.94E-13	2.40[1.92–3.00]	NA	NA	TNFAIP3	RA, CelD, UC, PS, SLE
rs1456893^S^	7	50.204	0.33	0.30	G	3.01E-03	3.20E-03	2.10E-02	1.14[1.05–1.25]	NA	NA	IKZF1	CD
rs10488631^M^	7	128.381	0.19	0.11	T	2.55E-27	2.71E-27	6.05E-25	1.97[1.74–2.22]	NA	NA	IRF5	RA, SLE, UC, SScl
rs2618476^SU^	8	11.390	0.30	0.25	T	1.10E-07	1.17E-07	4.34E-06	1.29[1.18–1.42]	NA	NA	BLK	SLE, RA
rs16940202^SMU^	16	84.572	0.09	0.02	T	4.76E-07	5.06E-07	1.41E-05	1.53[1.30–1.81]	NA	NA	IRF8	UC, MS
rs181359^M^	22	20.27	0.22	0.18	T	3.37E-06	3.58E-06	7.27E-05	1.28[1.15–1.41]	1.15E-09	1.23[1.15–1.33]	UBE2L3	CD, SLE

Only the most significant variant in each region is presented. All variants met a FDR-adjusted threshold of significance in the joint-analysis (FDR P<0.05), as described in the Materials and Methods. The genomic control-adjusted (GC) P-value is also shown. When both joint and LLAS1 results available, a meta-analysis between all cohorts is presented. The smallest *P-value* is presented and, unless noted otherwise, it is under the additive model. OR and CI calculated under the model presented.

Ch – chromosome; Mb – Megabases; MA – minor allele; MAF – Minor allele frequency; OR – odds ratio; CI – confidence interval; NA – not available.

*This marker failed quality control or was unavailable in the joint-analysis, here we are presenting the LLAS results.

1 The initials after the marker denote that this marker was imputed in a cohort: ^S^SLEGEN, ^M^MN, ^U^UCSF.

2 The superscript after the P-value denotes its genetic model, when other than the additive: ^d^dominant, ^r^recessive.

3 Diseases that reported any associated SNP in Caucasians with P-value<1.0×10^−5^ in the regions indicated (not necessarily the same SNP reported in this table) through the GWA approach [28].

Disease abbreviations are the same as for Table 1.

The *IL10* locus, which has not been previously reported in our independent SLE cohorts, has been reported in GWAS of T1D, BeD, CD and UC [28]. The rs3024493 variant, which was reported in two GWAS of UC, was significant in all cohorts (P_joint_ = 4.38×10^−05^). The reported risk allele is the same as we report. This is not a novel effect in SLE; although it has not been reported in a GWAS of SLE, *IL10* has been recently identified in a large-scale replication study [3]. This locus harbors a known copy number polymorphism/variation (CNV) (http://genome.ucsc.edu).

Similarly, the *IRF8* locus, which has not been previously reported in our independent SLE cohorts nor been reported in a GWAS of SLE, has also been identified in a large-scale replication study [3]. This region has been reported in GWAS of MS and UC [28]. The rs16940202 variant, which was reported in UC, showed association with SLE (P_joint_ = 4.76×10^−07^), but the alleles show opposing effects.

The *KIAA1109* region also showed association (rs13119723, P_meta_ = 4.97×10^−05^) (Table 3). This SNP was reported in a GWAS of RA, where the risk allele is consistent with our study of SLE [28]. Other SNPs in this region have also been reported in GWAS of T1D and CelD [28]. Interestingly, this region is adjacent to the *IL2–IL21* region, where several SNPs have been reported in GWAS of T1D, UC, CelD and AA. Variation in these regions has never been reported to have met genome-wide significance in SLE. Association has been reported in 200 Colombian patients for a SNP in LD with rs13119723 (rs6822844; r^2^ = 0.71) [30]. Association with SLE has also been reported in two studies of mixed ethnicities for other variants in IL2/*IL21* that lack LD (r^2^<0.30) with rs13119723 [31], [32].

For the vast majority of SNPs in Table 3, there is consistency between the SLE risk allele and that reported in other ADs. For example, the *IRF5* variant has the same risk allele as RA, and SScl, and the *TNFAIP3* variant has the same risk allele as in four different GWAS of RA [27]. There are two variants that exhibit opposite effects, namely in *FCGR2A* and *IKZF1*, with UC and CD, respectively [28].

### SLE–specific loci

In addition to significant associations in regions previously implicated in other ADs, we have also identified significant associations in established SLE regions that do not meet a GWA P<1.0×10^−5^ in other ADs. Table 4 shows the established SLE-specific variants and their association results in our combined cohorts. These variants have not been reported in GWAS of any AD except SLE, hence suggesting that these may be SLE-specific genetic risk factors or that their magnitude of effect in other ADs is more modest. Established SLE loci that are not strongly associated with other ADs include the *integrin-α_M_* (*ITGAM*), *tumor necrosis factor superfamily member OX40L* (*TNFSF4*), *pituitary tumor-transforming 1* (*PTTG1*), *PHD and ring finger domains 1* (*PHRF1*), *WDFY family member 4* (*WDFY4*), and *B-cell scaffold protein with ankyrin repeats 1* (*BANK1*) regions.

**Table 4:** SLE–specific loci: Association results for established SLE–specific regions in our joint cohorts.

					Joint-Analysis*					Meta-Analysis		
SNP^1^	Ch	Pos (Mb)	MAF case	MAF control	MA	P-value^2^	GC P-value	FDR P-value	OR [95%CI]	P-value^2^	OR [95%CI]	Region
rs10798269^M^	1	170.041	0.31	0.36	G	2.02E-05	2.15E-05	3.42E-04	0.83[0.76–0.90]	4.04E-10	0.83[0.78–0.88]	TNFSF4
rs10516487	4	103.108	0.27	0.32	G	4.88E-05	5.19E-05	6.81E-04	0.83[0.76–0.91]	NA	NA	BANK1
rs2313132	4	139.050	0.13	0.11	G	2.93E-03	3.12E-03	2.11E-02	1.21[1.07–1.38]	NA	NA	SLC7A11
rs2431697*	5	159.813	0.39	0.44	G	9.68E-08	1.03E-07	NA	0.80[0.74–0.87]	NA	NA	PTTG1
rs11101442^SU^	10	49.606	0.30	0.34	T	4.16E-05	4.42E-05	6.57E-04	0.83[0.76–0.91]	NA	NA	WDFY4
rs7927370^M^	11	54.893	0.04	0.06	T	1.13E-03	1.20E-03	1.12E-02	0.74[0.62–0.89]	NA	NA	OR4A51
rs4963128*	11	0.58	0.30	0.35	A	7.77E-07	8.26E-07	NA	0.81[0.74–0.88]	NA	NA	PHRF1
rs11150610	16	31.242	0.37	0.43	C	1.16E-08	1.23E-08	5.51E-07	0.79[0.72–0.85]	NA	NA	ITGAM

Only the most significant variant in each region is presented. All variants met a FDR-adjusted threshold of significance in the joint-analysis (FDR P<0.05), as described in the Materials and Methods. The genomic control-adjusted (GC) P-value is also shown. When both joint and LLAS1 results available, a meta-analysis between all cohorts is presented. The smallest *P-value* is presented and, unless noted otherwise, it is under the additive model. OR and CI calculated under the model presented.

Ch – chromosome; Mb – Megabases; MA – minor allele; MAF – Minor allele frequency; OR – odds ratio; CI – confidence interval; NA – not available.

*: This marker failed quality control or was unavailable in the joint-analysis, we are presenting the LLAS results.

1 The initials after the marker denote that this marker was imputed in a cohort: ^S^SLEGEN, ^M^MN, ^U^UCSF.

2 The superscript after the P-value denotes its genetic model, when other than the additive: ^d^dominant, ^r^recessive.

With the exception of *BANK1*, all of these effects have been previously reported based on individual analyses of the cohorts considered here.

### Autoimmune loci not associated with SLE

Our study was well powered to detect effect sizes (as measured by the odds ratio (OR)) similar to those reported in other ADs. Under the assumptions described in the Materials and Methods, we were powered to detect: OR>1.45 for variants with minor allele frequency (MAF) = 0.05, OR>1.30 for variants with MAF = 0.10, or OR>1.20 for variants with MAF>0.30. The power for each of the SNPs herein reported is shown in Table S1.

For many of the shared GWAS autoimmune loci we found no evidence for association with SLE in these cohorts. We scrutinized all variants that met QC criteria and whose smallest P-value was P>0.05 in any (joint-, replication- , or meta-) analysis. Amongst the loci shared between the most diseases, we found no evidence of association for *IL23R, FASLG, REL, IL18RAP, MST1, RBPJ, IL7R, PTGER4, BACH2, PVT1, PTPN2*, and *C1QTNF6* regions.

As in all studies a definitive answer is not possible for all loci. Specifically, we note that in several of the AD loci shared between the largest number of diseases, we did not find SNPs that met the FDR-adjusted threshold in the joint-analysis, but met all the QC criteria and had an unadjusted P-value<0.05: these include *SPRED2* (rs934734, P = 1.41×10^−02^), *AFF3* (rs10865035, P = 3.04×10^−02^), *CTLA4* (rs3087243, P = 1.66×10^−02^), *IL12B* (rs2082412, P = 1.02×10^−02^), *IL2RA* (rs12251307, P = 2.21×10^−02^), *LRRC32* (rs7927894, P = 2.32×10^−02^), *C14orf81* (rs4899260, P = 1.48×10^−02^), and *GSDMB* (rs8067378, P = 2.17×10^−02^). Further work is necessary to determine if these loci contribute modestly or conditionally to the risk of SLE.

### Shared autoimmune loci

**Figure 1:**
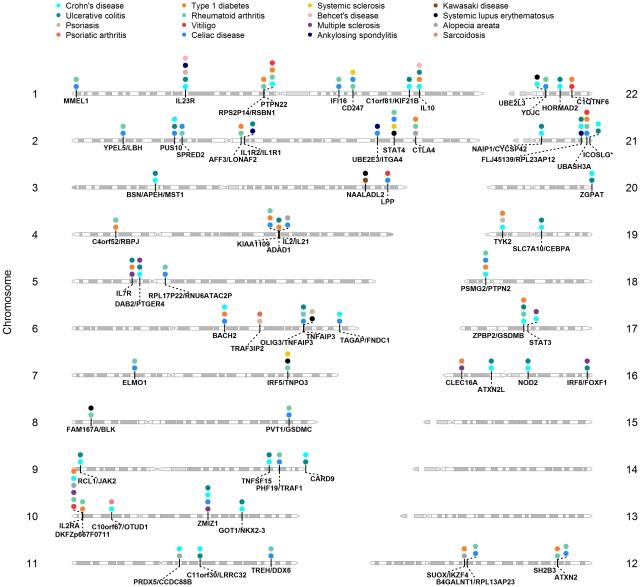
Shared *loci* between GWAS of ADs. Gene regions are below each chromosome, diseases where it was reported are above, represented by a colored circle.

An important goal of our study was to assess the extent of pleiotropy between all 17 ADs using their reported GWAS results. Figure 1 shows the loci shared between GWAS of ADs. The regions shared across the largest number of ADs include *IL2RA* (MS, T1D, RA, VI, AA and CD), *IL23R* (CD, UC, PS, BeD and AS), *OLIG3/TNFAIP3* (RA, PS, SLE, UC, and CelD), *PTPN22* (CD, RA, T1D, and VI), *IL10* (CD, T1D, UC, and BeD), and an intergenic region between *ZPBP2* and *GSDMB* (CD, RA, UC, and T1D).

**Figure 2:**
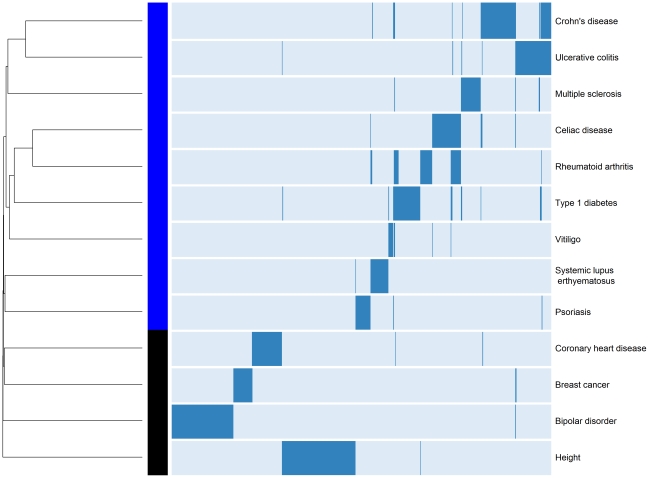
Hierarchical clustering of ADs and four non-ADs. Analysis was restricted to diseases with >10 reported *loci* in their GWA studies, to associations reported from populations of European ancestry, and excluded those reported from the aggregate phenotype of IBD, as well as those with MS severity or age of onset. Dendrogram and heatmap are shown.

We also sought to better understand the relationships among different diseases given the common and unique regions reported in their respective GWAS. In order to identify which diseases cluster together based on the reported shared regions (i.e., binary yes/no), we performed hierarchical clustering analysis of ADs also including reported risk loci from 4 control traits/diseases including height, breast cancer, coronary heart disease, and bipolar disorder (Figure 2). This analysis provides a visual characterization of the similarities among diseases based on the number of shared regions. The diseases that share the largest number of genomic regions include CD and UC (*BSN/APEH/MST1, C11orf30/LRRC32, C1orf81/KIF21B, CARD9, DAB2/PTGER4, GOT1/NKX2-3, IL10, IL23R, NAIP1/CYCSP42, PUS10, RCL1/JAK2, TNFSF15, ZGPAT, ZPBP2/GSDMB*), followed by T1D and RA (*AFF3/LONAF2, C4orf52/RBPJ, CTLA4, DKFZp667F0711, IL2RA, KIAA1109, PTPN22, RPS2P14/RSBN1, SH2B3/ATXN2, UBASH3A, ZPBP2/GSDMB*).

We observed that SLE shares the largest number of loci with RA (*FAM167A/BLK, IRF5/TNP03, and STAT4*). It is noteworthy that SLE appears isolated from the other ADs (i.e., shares the least with the other ADs despite being among the ADs with the most identified risk loci). In contrast, despite only having 12 reported loci, VI clusters more closely with other ADs, suggesting more genetic overlap. However, we strongly caution against over-interpretation of this clustering result, as bootstrapping only revealed strong statistical support (Bootstrap Probability Value≥0.95) for differentiating height from the ADs and the other control diseases (see Materials and Methods).

## Discussion

The clustering of multiple autoimmune disorders in families and evidence for autoimmune pleiotropic loci are well known. Nevertheless, no comprehensive assessment of the specific shared variants between SLE and other autoimmune diseases (ADs) has yet been performed in a single large-scale study based on GWAS data. Analyses of shared SLE loci have been limited to specific loci and few diseases (reviewed in [12]). In this study we used findings from published GWAS to assess the extent of genetic overlap between SLE and seventeen autoimmune diseases, testing if variants implicated in other ADs show association in our large SLE cohort. Given that the MHC is unquestionably a universal risk region for autoimmunity, and some GWAS did not report their results in this region, we excluded HLA loci from our analyses.

The loci that were associated with the largest number of ADs include *IL23R*, *TNFAIP3*, and *IL2RA*, supporting an important role for T cell and innate immune response pathways in autoimmunity. Nevertheless, these loci are not implicated in all ADs, suggesting that, with the exception of the HLA region, there seem to be no universal genetic risk factors for autoimmunity. It is commonly accepted that there is a common genetic background predisposing to autoimmunity and inflammation, and that further combinations of more disease-specific variation at HLA and non-HLA genes, in interaction with epigenetic and environmental factors, contribute to disease and its clinical manifestations [33]. Our data additionally suggests that, instead of resulting from common risk factors, autoimmunity may result from specific and multiple different pleiotropic effects. This is consistent with a recent report showing that genomic pleiotropy is relatively low, as most genes affect only a small number of traits [34]. The authors suggest that genes displaying a high degree of pleiotropy also exhibit an individually larger effect on each trait [34]. It is likely that different population genetic factors (e.g., natural selection, migration/isolation, random mutation) in similar or distinct environments led to the establishment of different autoimmune loci and subsequent migrations and interbreeding have led to the current plethora of loci that predispose to autoimmunity.

Based on our analyses of shared non-HLA loci across ADs, the most genetically similar diseases appear to be CD with UC, and T1D with RA, sharing 15 and 11 loci, respectively. While the former pair is clearly supported by overlapping clinical manifestations, since both CD and UC are subsets of IBD, the overlap between the latter pair is not entirely clear based on their organ involvement. The clustering patterns do not seem biased by the number of reported loci for each disease. As such, while the genetic overlap between CD and UC may reflect the prevalence of more specific IBD genes, the genetic overlap between T1D and RA may reflect the existence of general, nonspecific autoimmunity genes.

Despite being a prototypic AD, the non-HLA genetic overlap between SLE and the ADs herein investigated is more modest than we anticipated. The disease with which it shares the most loci is RA, which is potentially interesting due to the common clinical presentation of arthritis. The number of reported SLE loci is similar to other ADs and does not explain its relative distance from other ADs. The clinical heterogeneity of SLE may, at least in part, account for the relatively modest number of shared loci. Different SLE loci are likely differentially associated with specific clinical criteria, as was recently shown in GWAS of anti-RNA binding proteins [35], and anti–dsDNA autoantibody production [36] in SLE. It should also be noted that SLE may share more loci with systemic diseases not included or not well represented in our analyses. Our data included 49 loci reported for RA, two for BeD, three for SScl, but Sjögren's syndrome and antiphospholipid syndrome lack GWAS. Interestingly, two of the three loci reported in the GWAS of SScl, *IRF5* and *STAT4*, also show association in GWAS of SLE. Similarly, Anaya *et al.* [37] recently analyzed the association of the SLE predisposing risk variant (rs1143679) for *ITGAM-ITGAX* across 7 other ADs, only showing a suggestive association for SScl. For many of the shared GWAS autoimmune loci we found no evidence for association with SLE, including for *IL23R*, in spite of having enough power to detect the effects reported in other diseases. Although we cannot exclude the possibility that 1) other variants in these loci predispose to SLE, or 2) that these loci have weaker effects in SLE implying a potential lack of statistical power, or 3) that their effects are conditional on other unknown loci, it is plausible that the lack of these common genetic factors contributes to SLE being a distinct disease. Also, several established SLE loci are apparently not associated with other ADs, including the *ITGAM-ITGAX* region, *TNFSF4*, *PTTG1*, *PHRF1*, *WDFY4* and *BANK1* regions. Obviously, these risk variants may simply have weaker effects in other ADs and the studies lacked power to detect them. This situation was recently illustrated in a meta-analysis of CD and CelD, where the increased power of the combined datasets allowed the detection of shared loci with a relatively small effect, hence undetectable in the individual diseases [22].

Our analyses identify novel shared SLE loci. The results that we report were adjusted for the number of comparisons, which decreases the likelihood of a false positive result. The *V-set domain containing T cell activation inhibitor 1* (*VTCN1*) region, which has been reported in a GWAS of JIA, showed the strongest novel association with SLE. Evidence suggests that this gene plays a role in the negative regulation of T cell responses. The zinc finger *ZGPAT* region also shows a significant association with SLE. Despite being clearly strong candidates because of their association with other ADs, the new SLE loci require validation. It is worth noting that we discovered associations consistent with and in contrast to the same risk allele in other ADs. This observation was recently confirmed by Wang et al. [34], who suggests that susceptibility loci involved in the pathogenesis of ADs may have antagonistic pleiotropic effects, where risk alleles for one disease may confer selective advantage for another disease or infection resistance. Given that the functional variant is not known, we cannot rule out that the inverse association arises from different LD patterns.

A limitation of our study is the fact that we restricted our analyses to variants reported from GWAS in populations of European Ancestry. Although we have certainly missed shared variants identified in large candidate gene studies or targeted meta-analyses, many ADs lack such studies. Thus, given the increasing coverage of the genome with modern SNP chips, we preferred to restrict our analyses to a directly comparable set of results based on GWAS. These agnostic scans help to minimize the extent of potential methodological and publication biases. We should note that our analyses do not provide an unbiased estimate of the total degree of genetic overlap amongst ADs, given that the application of stringent significance thresholds in GWAS certainly overlooks true risk loci. Future studies using all variants in these GWAS will be required to directly estimate the degree of shared susceptibility. Finally, it is important to note that some of the genetic overlap with SLE may have been missed in our analyses because a large proportion of candidate SNPs failed our quality control thresholds, and thus could not be effectively tested for association in our samples.

Much remains to be done before the genetic etiology of the autoimmunity spectrum is resolved. Continued studies of populations beyond those of European ancestry are certainly needed. A catalog of all shared and distinct risk loci requires that these regions be thoroughly resequenced in suitably large population samples, with additional genotyping of the resulting comprehensive set of variants in order to confirm and fully characterize the extent of genetic risk. The examination of the patterns observed here generates an appreciation for potential interplay between population genetic factors (e.g., natural selection, migration) and environmental factors and calls for the interrogation of these loci in significant numbers of samples from different ethnic populations.

This study represents the most comprehensive evaluation of shared autoimmune loci to date. In addition, we provide further evidence for previously and newly identified pleiotropic genes in SLE. These findings support a relatively distinct genetic susceptibility for SLE, a genetic basis for the shared pathogenesis of ADs, and the value of studies of potentially pleiotropic genes in autoimmune diseases.

## Materials and Methods

### Ethics statement

Written informed consent was obtained from all study participants and the institutional review board at each collaborating center approved the study.

### Autoimmune disease loci

We constructed a list of reported risk variants for ADs using data from the National Human Genome Research Institute's Catalog of Published Genome-Wide Association Studies (http://www.genome.gov/gwastudies) accessed on June 4^th^, 2011 [28]. Briefly, this database contains all identified SNPs with P<1.0×10^−5^ from GWAS that attempted to assay at least 100,000 SNPs in the initial analysis stage, thus excluding studies focused on candidate genes. We extracted any reported risk variants for the following ADs: SLE, rheumatoid arthritis (RA), type 1 diabetes (T1D), ankylosing spondylitis (AS), Crohn's disease (CD), ulcerative colitis (UC), celiac disease (CelD), multiple sclerosis (MS), systemic sclerosis (SScl), psoriasis (PS), psoriatic arthritis (PsA), juvenile rheumatoid arthritis (JIA), Kawasaki disease (KA), sarcoidosis (SA), vitiligo (VI), alopecia areata (AA) and Behçet's disease (BeD). Given that not all of these GWAS reported their Major Histocompatibility Complex (MHC) or Human Leukocyte Antigen (HLA) results, we opted for excluding this region, in order to avoid biases due to missing HLA data from some studies. We excluded inflammatory bowel disease and MS severity or age of onset, and have only included results published in samples of European ancestry. After removing SNPs that map to the MHC and those with reported associations based on haplotypes, our final list of reported risk variants included 446 SNPs. We then mapped these SNPs to genomic regions using a block partitioning approach [38], based on LD-information estimated from the CEU (Utah residents with ancestry from northern and western Europe) sample of the International HapMap Project.

### Clinical samples

Samples used in this study have been previously described [24]–[27]. Briefly, we combined 743 SLE cases and 3566 controls from the International Consortium for Systemic Lupus Erythematosus Genetics (SLEGEN; www.slegen.org) GWAS [25], with 244 SLE cases and 2140 controls from the Minnesota (MN) cohort GWAS [24], and with 513 SLE cases from the University of California San Francisco (UCSF) Lupus Genetics Project [26], [27]. These 513 cases were included and are also described in a GWAS of SLE [39]. Exclusion of duplicates and first-degree relatives yielded a total sample of 1500 cases and 5706 controls. Quality control was performed as described [25]. We included males and females of European ancestry.

In addition, we used data from the Lupus Large Association Studies (LLAS) [25], when available, as a replication cohort. The LLAS replication study consists of an independent cohort of 2085 SLE cases and 2854 controls, and was used to replicate 8230 SNPs from the SLEGEN GWAS [25]. This study consists of males and females of European ancestry.

### Statistical analysis

Genotypes from all subjects were imputed using the program IMPUTE [40] version 0.5 for SNPs not genotyped or poorly genotyped. Imputation was performed using high quality genotype data from the corresponding study (SLEGEN, MN and UCSF) and phased HapMap Phase II (NCBI B35 assembly) genotype data from 60 CEU HapMap founders. We used SNPs that met the following quality criteria: 1) no statistically significant differences in the proportions of missing genotype data between cases and controls (i.e., *P*>0.05); 2) overall <10% missing genotype data; 3) Hardy-Weinberg Expectations (HWE) in controls *P*>0.01, HWE in cases *P*>0.0001; and 4) minor allele frequencies (MAFs) of controls within a 95% or 99.99% confidence interval for ethnicity matched HapMap MAFs, for genotyped and imputed SNPs, respectively. Retained SNPs had an estimated MAF>0.01 in the control samples, an information score >0.50 and a confidence score >0.90. Imputed SNPs were analyzed using SNPTEST with probabilistic genotypes [40].

We combined the genotypic and imputed data from the three cohorts described above and performed a joint- and a meta-analysis. In the tables with the results we report which SNPs and cohorts were imputed vs. directly genotyped. We used SNPs that met the same quality criteria as described above. To account for potential population stratification, we computed Principal Components (PCs) and adjusted these analyses for four PCs, as described [25]. The genome-wide inflation factor in the joint analysis was λ = 1.15. We include the joint analysis of these loci after applying quality control to each individual cohort as the joint analysis can provide increased power for some genetic models for more modest allele frequencies (e.g., recessive model). From our list with 446 autoimmune SNPs, 424 total unique SNPs were genotyped or imputed in our SLE cohorts. Of these, 237 (55.9%) met our QC thresholds, while 187 (44.1%) failed as follows: 6 (1.4%) have 10–20% missing genotype data, 1 (0.2%) have MAF<0.01 in controls, 3 (0.7%) failed Hardy-Weinberg Equilibrium thresholds, 91 (21.5%) have >20% missing genotype data, and /or have significant differences in missingness between cases and controls, and 86 (20.3%) did not meet imputation QC thresholds. We report uncorrected *P-values*, though we also corrected for multiple comparisons using a False Discovery Rate (FDR) procedure [29] for the 237 SNPs that passed QC. As such, our multiple comparison strategy consisted of only selecting those variants that met FDR significance, that is, with a FDR-adjusted P-value<0.05. Although we computed the FDR-adjusted P-value for the smallest P-value (under the additive, dominant or recessive model), this smallest P-value is virtually always within one order of magnitude different from the additive P-value, which is hence comparable to computing the FDR for P-values under the same model. We performed a weighted Z-score meta-analysis as implemented in METAL (www.sph.umich.edu/csg/abecasis/metal), with weights being the square root of the sample size for each dataset; thus, the meta-analysis incorporates direction, magnitude of association and sample size. We report the minimum *P-value* based on hypothesis tests considering additive, dominant and recessive modes of inheritance; however, because these tests can be affected by low genotype counts, we required at least 30 homozygotes for the minor allele to consider the recessive, and 15 to consider the additive model, otherwise the results under the dominant model are reported. All genetic models were defined relative to the minor allele. Associations with SLE susceptibility were considered statistically significant if they met a FDR-adjusted threshold of *P*<0.05.

We used Quanto (http://hydra.usc.edu/gxe/) to calculate the power of our sample size. We assumed an additive genetic model, population risk of 0.1%, and α = 0.001.

In order to examine the global similarity between ADs based on their reported risk loci (defined based on LD, as described above), we performed a hierarchical clustering analysis of ADs with at least 10 reported loci (binary yes/no). ADs with less than 10 reported loci were excluded as their lower count of reported loci may reflect a less intensive assessment of genetic risk factors (i.e. fewer genome-wide investigations often with smaller sample sizes). We restricted the analysis to associations reported from populations of European ancestry, and excluded those reported for MS severity or age of onset. So as not to inform the clustering of ADs based on the presence of joint analyses, we excluded associations from studies of pooled phenotypes including IBD, RA with CelD, CD with CelD, and CD with SA. This produced a final dataset of 330 loci reported across nine ADs. We also included loci reported from the GWAS catalogue for 4 control diseases (height, breast cancer, coronary heart disease, and bipolar disorder), similarly using LD to define specific genomic loci. We computed the dissimilarity between ADs and the control diseases using distance metric appropriate for binary data, performing hierarchical clustering using the *hclust* function for the R Statistical Programming Language [41]. We evaluated the uncertainty in the clustering analyses using a multiscale bootstrap resampling approach implemented within the *pvclust* package for R [42].

## Supporting Information

Table S1 First tier shows the SNPs presented in Table 2 of the manuscript, followed by Table 3 in the middle and Table 4 at the bottom. The power was computed in the joint-analysis of 1,500 cases and 5,706 controls, under the genetic model presented, assuming a population risk of 0.1% and α = 0.001. OR – odds ratio; CI – confidence interval; MAF – Minor allele frequency. The smallest *P-value* is presented and, unless noted otherwise, it is under the additive model. OR and CI calculated under the model presented. * The superscript after the P-value denotes its genetic model, when other than the additive: ^d^dominant, ^r^recessive.
(DOC)
