# Supplementary material for: A Comprehensive Analysis of Shared Loci between Systemic Lupus Erythematosus (SLE) and Sixteen Autoimmune Diseases Reveals Limited Genetic Overlap
Source: PLoS Genet. 2011 Dec 8;7(12):e1002406. doi: 10.1371/journal.pgen.1002406 (PMC3234215; doi:10.1371/journal.pgen.1002406)
Supplement: Table S1 — First tier shows the SNPs presented in Table 2 of the manuscript, followed by Table 3 in the middle and Table 4 at the bottom. The power was computed in the joint-analysis of 1,500 cases and 5,706 controls, under the genetic model presented, assuming a population risk of 0.1% and α = 0.001. OR – odds ratio; CI – confidence interval; MAF – Minor allele frequency. The smallest P-value is presented and, unless noted otherwise, it is under the additive model. OR and CI calculated under the model presented. * The superscript after the P-value denotes its genetic model, when other than the additive: ddominant, rrecessive. (DOC) [file pgen.1002406.s001.doc]

Table S1. Estimates of power for the reported SNPs.

| SNP | Region | P-value* | OR [95%CI] | MAF | Power |
| --- | --- | --- | --- | --- | --- |
| rs12046117 | VTCN1 | 2.02E-06^d^ | 1.65[1.34-2.03] | 0.02 | 0.77 |
| rs6738825 | PLCL1 | 3.12E-03^d^ | 0.82[0.72-0.94] | 0.47 | 0.44 |
| rs17810546 | IL12A | 2.47E-03 | 0.82[0.72-0.93] | 0.11 | 0.33 |
| rs7672826 | RPL19P8 | 1.90E-03^d^ | 0.83[0.74-0.93] | 0.33 | 0.47 |
| rs881375 | VEGFA | 3.89E-03 | 1.14[1.04-1.24] | 0.34 | 0.41 |
| rs1953126 | TRAF1 | 2.93E-03^d^ | 1.20[1.06-1.35] | 0.34 | 0.42 |
| rs7221109 | CCR7 | 2.21E-03^d^ | 1.21[1.07-1.36] | 0.39 | 0.44 |
| rs6074022 | CD40 | 1.41E-03^r^ | 1.45[1.15-1.82] | 0.26 | 0.61 |
| rs2297441 | ZGPAT | 7.63E-04 | 0.84[0.76-0.93] | 0.22 | 0.56 |
| rs3024493 | IL10 | 4.38E-05 | 1.26[1.13-1.41] | 0.16 | 0.84 |
| rs6445975 | PXK | 4.51E-04 | 1.18[1.07-1.29] | 0.30 | 0.68 |
| rs13119723 | KIAA1109 | 5.56E-03^d^ | 0.82[0.71-0.94] | 0.13 | 0.32 |
| rs11747270 | IRGM | 1.12E-03^d^ | 1.39[1.14-1.70] | 0.03 | 0.29 |
| rs5029939 | TNFAIP3 | 1.51E-14^d^ | 2.40[1.92-3.00] | 0.04 | 0.99 |
| rs1456893 | IKZF1 | 3.01E-03 | 1.14[1.05-1.25] | 0.31 | 0.38 |
| rs10488631 | IRF5 | 2.55E-27 | 1.97[1.74-2.22] | 0.13 | 0.99 |
| rs2618476 | BLK | 1.10E-07 | 1.29[1.18-1.42] | 0.26 | 0.99 |
| rs16940202 | IRF8 | 4.76E-07 | 1.53[1.30-1.81] | 0.03 | 0.75 |
| rs181359 | UBE2L3 | 3.37E-06 | 1.28[1.15-1.41] | 0.19 | 0.47 |
| rs10798269 | TNFSF4 | 2.02E-05 | 0.83[0.76-0.90] | 0.35 | 0.83 |
| rs10516487 | BANK1 | 4.88E-05 | 0.83[0.76-0.91] | 0.31 | 0.79 |
| rs2313132 | SLC7A11 | 2.93E-03 | 1.21[1.07-1.38] | 0.11 | 0.47 |
| rs11101442 | WDFY4 | 4.16E-05 | 0.83[0.76-0.91] | 0.33 | 0.82 |
| rs7927370 | OR4A51 | 1.13E-03 | 0.74[0.62-0.89] | 0.06 | 0.43 |
| rs11150610 | ITGAM | 1.16E-08 | 0.79[0.72-0.85] | 0.42 | 0.99 |
